# Level of phospho-STAT3 (Tyr705) correlates with copy number and physical state of human papillomavirus 16 genome in cervical precancer and cancer lesions

**DOI:** 10.1371/journal.pone.0222089

**Published:** 2019-09-05

**Authors:** Shirish Shukla, Mohit Jadli, Kulbhushan Thakur, Gauri Shishodia, Sutapa Mahata, Seemi Farhat Basir, Bhudev Chandra Das, Alok Chandra Bharti

**Affiliations:** 1 Division of Molecular Oncology, National Institute of Cancer Prevention and Research Noida, Uttar Pradesh, India; 2 Molecular Oncology Laboratory, Department of Zoology, University of Delhi, Delhi, India; 3 Department of Biosciences, Jamia Millia Islamia, New Delhi, India; 4 Amity Institute of Molecular Medicine and Stem Cell Research (AIMMSCR) Amity University, Noida, Uttar Pradesh, India; Istituto Nazionale Tumori IRCCS Fondazione Pascale, ITALY

## Abstract

Our earlier studies indicated an important role of inducible transcription factor STAT3 in the establishment of persistent infection of human papillomavirus (HPV) type 16 and promotion of cervical carcinogenesis. Since HPV load and its physical state are two potential determinants of this virally-induced carcinogensis, though with some exceptions, we extended our study to examine the role of active STAT3 level in cervical precancer and cancer lesions and it’s association with HPV viral load and physical state. An elevated level of active STAT3 was measured by assessing phospho-STAT3-Y705 (pSTAT3), in tumor tissues harboring higher viral load irrespective of the disease grade. Physical state analysis of HPV16 by assessing the degree of amplification of full length E2 and comparing it with E6 (E2:E6 ratio), which predominantly represent episomal form of HPV16, revealed low or undetectable pSTAT3. A strong pSTAT3 immunoreactivity was found in tissues those harbored either mixed or predominantly integrated form of viral genome. Cumulative analysis of pSTAT3 expression, viral load and physical state demonstrated a direct correlation between pSTAT3 expression, viral load and physical state of HPV. The study suggests that there exists a strong clinical correlation between level of active STAT3 expression and HPV genome copy number, and integrated state of the virus that may play a pivotal role in promotion/maintanence of tumorigenic phenotype.

## Introduction

Progression to cervical cancer is a multi-step process etiologically-linked with persistent infection of high-risk human papillomaviruses (HPVs). Though HPV infection is a necessary prerequisite, but it is not sufficient for the initiation of cervical cancer [1]. The physical state of the viral genome (integrated vs. episomal) and viral copy number in infected tissues have been evaluated as the candidate surrogate markers for the early detection of high grade and potentially progressive lesions, which showed a predictive potential of these biomarkers [2–4]. Viral integration often leads to the disruption of its E2 gene [5]. Loss of functional E2 gene abolishes the transcription-repressive effect of E2 on the expression of viral oncogenes E6 and E7 [6]. Absence of E2 due to insertional inactivation or by epigenetic silencing [7] and expression of E6 and E7 collectively drive the process of carcinogenesis [8,9]. On the contrary, integration of the HPV genome in low-grade lesions and occasionally in normal cervical tissues has also been reported [10,11], whereas, not all invasive cancers carry the integrated HPV genome [12–14]. Clinical implication and the reasons behind such discrepant observations, particularly the confounding factors responsible for the phenomenon, are not clear as yet. Despite these variations, high-risk HPV viral load and physical state are proposed as potentially useful markers that could predict progressive high-grade cervical lesions [15–17]. These markers, however, display differential type-dependent risks [18]. Nevertheless, in the case of HPV16 infection, these parameters (viral load and integration) consistently showed increased risk [4,19]. Therefore, a better understanding of factors that control these viral attributes will improve the performance of viral infection-specific biomarkers in predicting cervical disease progression, and subsequently the therapeutic outcome.

Expression of HPV genome depends primarily on host transcription factors that work on specific enhancer regions present in HPV Upstream Regulatory or Long Control Region (URR/LCR) [20]. A set of transcription factors like STAT3, AP-1, NF-κB, SP1, NF-1, c/EBP, Oct-1, KRF-1, YY1, and GRE have been proposed to play a regulatory role in HPV infection due to the presence of their cognate *cis*-elements in the URR [20–22]. These factors are responsible for the cell-type-specific viral gene expression and contribute to the tissue tropism of HPVs [20,23]. Apart from critically influencing the viral oncogenes expression, host transcription factors directly influence other important determinants of HPV infection such as viral replication [24,25] and may be involved in viral integration in host cell genome via induction of genomic instability [26]. Characterization of host cell transcription factors has revealed a disease stage and grade-specific expression and activity of some of the key transcription factors like AP-1, NF-κB, and STAT3 in cervical cancer carcinogenesis [27–29]. However, any influence/association of these transcription factors on the maintenance of the HPV genome in an infected cell remains elusive. Therefore, improved understanding of molecular dynamics of specific host transcription factors expression and their correlation with the HPV genome physical state and copy number in cervical carcinogenesis is required to develop effective infection-specific biomarkers and the therapeutic targets.

Assessment of promoter activity in HPV16 [30] and ChIP-sequencing data of HPV18 [22] revealed presence of binding sites for STAT3 in URR, which plays a pivotal role in epithelial carcinogenesis [31]. STAT3 expression and activation is known to increase with disease severity [29] and has been shown to contribute functionally by regulating expression of viral oncogene E6 [32]. However, the functional relevance of STAT3 concerning the viral load and physical state of the HPV genome in the host cell remained unexplored. STAT3 is an inducible transcription factor that works as an important link between inflammation and carcinogenesis [33]. Its activity and nuclear translocation are controlled by specific phosphorylation at tyrosine 705 [pSTAT3(Y705)] that results in its dimerization, nuclear translocation, and DNA binding [33]. Evidence from our group [34] and others [35], indicate the presence of active STAT3 in cervical cancer stem cells. A recent study carried out on keratinocytes harboring HPV18 episomes demonstrated an essential role of active STAT3 in the maintenance of viral genome [36]. However, a clinical correlation of this experimental observation was lacking.

In the present study, we investigated the existence of a correlation between active STAT3 and physical state as well as the copy number of viral genome in HPV16 positive tumor tissues from cervical precancer and cancer lesions. Level of active STAT3 (pSTAT3) was measured by immunoblotting of total proteins isolated from different cytopathological grades of tumor tissues. In parallel, DNA isolated from the respective tissues was subjected to analysis of HPV16 viral load which was measured by the copy number analysis, and the physical state was examined by measuring the ratio of amplification of E2 versus E6 gene regions in HPV16 genome. To maintain homogeneity of the analysis, and to avoid confounding variables, cervical precancer and cancer tissues having non-HPV16 or multiple infections were excluded from the study.

## Materials and methods

### Ethics statement

A total of 252 fresh cervical biopsies were collected prospectively comprising of cervical tissues before any chemo-/radio-therapy from the Cancer Clinic, Gynae Out Patient Department of Lok Nayak Hospital, New Delhi, India. The age of participants ranged between 23 to 80 years. Written informed consent was obtained from all the participants included in the study and was carried out by the Principles of the Helsinki Declaration, and clinico-epidemiological details were taken from their clinical records. The study was approved by the Institutional Ethics Committee of the Institute of Cytology and Preventive Oncology (now renamed as National Institute of Cancer Prevention and Research), Noida, Utter Pradesh, India.

### Clinical specimens and reagents

Out of 252 specimens, 130 HPV16 positive cervical tissues consisting of 60 pre-cancers with abnormal cytopathological diagnosis [LSIL (30) or HSIL (30)], and 70 cancer tissues qualified for analysis of viral load and the physical state of HPV16 by PCR-based method and pSTAT3 expression using western blotting (**Table 1**). A portion of each biopsy collected in cold 1X phosphate buffer saline (PBS) was immediately processed for molecular research work, and the other half was sent for routine histopathological diagnosis in formalin solution. All reagents used in the study were of analytical or molecular biology grade and procured from Sigma Aldrich (USA) unless specified. Custom-synthesized, HPLC-purified primers were procured from either M/s Microsynth (Germany) or M/s Eurogentec (Belgium). Primers used in the study are listed in **Table 2**[37–39].

**Table 1:** Clinicopathological distribution of subjects enrolled in the study along with PCR-based analysis of HPV infection in their tumor tissues.

Tissue type	Diagnosis	Number of samples	Total HPV+ (HPV L1) (n/%age)	HPV 16* (n/%age)	HPV18* (n/%age)	Others HPV (n/%age)	Multiple infections	Mean age (Years; ± SD)	No. of HPV16 positive cases qualified
**Normal**		**32**	**2 (6%)**	**2 (6%)**	**-**	**-**	**-**	**40.5 ± 8.2**	**-**
**Pre-cancer**		**120**	**65 (54%)**	**60 (50%)**	3 (2.5%)	2 (1.5%)	**-**	**37.4 ± 6.9**	**60**
	LSIL	70	33 (47%)	30 (43%)	2 (3%)	1 (1.5%)	-		30
	HSIL	50	32 (64%)	30 (60%)	1 (2%)	1 (2%)	-		30
**Cancer**		**100**	**96 (96%)**	**89 (89%)**	**7 (7%)**	**3 (3%)**	4*(4%)	**51 ± 11.8**	**70**
***Histopathological grading***	WDSCC	55	52 (95%)	46 (84%)	5 (9%)	2 (4%)	2 (4%)		35
	MDSCC	35	34 (97%)	33 (94%)	2 (9%)	1 (3%)	2 (6%)		25
	PDSCC	10	10 (100%)	10 (100%)	-	-	-		10

*Includes 3 cases of multiple infections of HPV16 with HPV18. HPV- human papillomavirus, SCC- squamous cell carcinoma, LSIL- low grade squamous intraepithelial lesions, HSIL-high grade squamous intraepithelial lesions, WDSCC-well differentiated SCC, MDSCC-moderately differentiated SCC, PDSCC-poorly differentiated SCC.

**Table 2:** Oligonucleotide primers used for HPV diagnosis, HPV16 typing, viral load quantitation (HPV16 URR) and E2: E6 PCR in the present study.

Primer	Nucleotide Position in HPV	Amplicon size (bp)	Primers Type	Primer sequences	Final Conc. (μM)	References
**MY 09 and**	L1 Consensus	450	*Forward*	5’CGT CCM ARR GGA WAC TGATC-3’	20 pmoles	[39]
**MY 11**			*Reverse*	5’-GCM CAG GGW CAT AAY AAT GC-3’		
				(M = A+C, W = A+T, Y = C+T, R = A+G)		
**HPV16URR**	7763–7781	217	*Forward*	5’-AAG GCC AAC TAA ATG TCA C-3’	20 pmoles	[37]
	57–75		*Reverse*	5’-CTG CTT TTA TAC AA CCG G-3’		
**p53 Exon 5**	463–482	184	*Forward*	5’-TAC TCC CCT GCC CTC AAC AA-3’	20 pmoles	[37]
	534–562		*Reverse*	5’-CAT CGC TAT CTG AGC AGC GC-3’		
**HPV16 E2**	2734~2753	1139	*Forward*	5'-AGG ACG AGG ACA AGG AAA A-3'	20 pmoles	[37]
	3853–3872		*Reverse*	5'-GGA TGC AGT ATC AAG ATT TG-3'		
**HPV 16 E6**	83–102	477	*Forward*	5’-GAA ACC GGT TAG TAT AAA AGC AGA C-3’	20 pmoles	[38]
	540–559		*Reverse*	5’- AGC TGG GTT TCT CTA CGT GTT CT-3’		
**β-Globin**		268	*Forward*	5’-GAA GAG CCA AGG ACA GGT AC-3’	10 pmoles	[37]
			*Reverse*	5’-CAA CTT CAT CCA CGT TAC ACC-3’		

### DNA extraction and diagnosis of HPV infection

High molecular weight genomic DNA was isolated from precancerous and cancerous cervical biopsies by the standard phenol-chloroform and proteinase K digestion procedure. PCR amplification was performed following the procedure described earlier [29]. The initial HPV diagnosis was performed by using a pair of L1 consensus degenerate primers (MY09 and MY11) based PCR method described earlier. HPV16 typing was done by type-specific primers (**Table 2**). Subsequently, HPV16 positive samples were subjected to comprehensive HPV genotyping by PGMY-Reverse Line Blot, which can detect about 32 HPV types including all high-risk and low-risk types [40] and samples with only monotypic HPV infection were included in the study.

### HPV16 viral load determination by real-time quantitative PCR (qRT-PCR)

Quantification of HPV16 viral copy number and measurement of input cellular DNA copies was performed as described earlier [2] with an iQ-Cycler system (Biorad, Hercules, CA, USA) using a recommended iQ SYBR green PCR supermix according to the manufacturer’s instruction. HPV16 URR primers were used for HPV copy number calculation, and p53 exon5 primers were used for input DNA copy number calculation. Since the amplicon size of p53 exon5 primer set was much closer to the HPV16 URR PCR product and had similar efficiency of amplification; we used this primer set to control host DNA input. Pre-calibrated WHO’s HPV16 International Standard DNA (06/202) procured from National Institute of Biological Standards and Control (NIBSC), UK was used as a reference. Standard curves used to quantify HPV16 copy number were made with ten fold serial dilutions of the WHO HPV16 international standard containing 50,000, 5000, 500, 50 and 5, HPV16 DNA copies diluted in the background of C33a genomic DNA. Briefly, the reaction was performed in a final volume of 25μl containing 1X SYBR green super-mix with 0.25μM of HPV16 URR forward and reverse primers and 50ng of genomic DNA of test samples. The URR primers were selected for viral load quantitation as these are retained in both episomal and integrated forms of the HPV16 viral genome. The PCR amplification was performed as follows: 1 cycle of 96°C for 3min, 40 cycles at 94°C for 30sec, 55°C for 30sec and 72°C for 30sec with realtime measurement performed during amplification step (72°C) at each cycle. Each real-time amplification was followed by a melt curve analysis for confirmation of predicted amplicon. Crude viral load or copy number of HPV16 genome in the clinical sample was calculated by the interpolation of standard curves of the dilution series generated by the Sequence Detection Software (iCycler iQ software version 3.0) of iCycler iQ real-time PCR detection system (Biorad, Hercules, USA). On the other hand, samples with viral loads higher than 50,000 copies/reaction were diluted in water to bring it down to the range of the standard curve. The viral load values were normalized to input host diploid genomic DNA using p53 exon5 amplification calibrated with C33a genomic DNA standard (NIBSC) as indicated below:

#### Normalized HPV16 viral load/unit host cell genome = HPV16 URR copy number/ number of diploid host genomes

The normalized HPV16 viral copy numbers are expressed as the number of viral copies/unit host cell genome.

### Determination of physical state of HPV16 genome in cervical tissues

The physical state of HPV16 genome was determined by PCR as described previously [2]. To determine HPV16 physical state, primers for full-length HPV16 E2 validated by our laboratory previously [37], were utilized to analyze the presence of intact E2 ORF that is disrupted or deleted in the integrated virus. Amplification of HPV16 E6, which is invariably retained in the integrated virus, was used as the denominator of total HPV16 DNA irrespective of its physical state of the virus. Briefly, genomic DNA of HPV16-positive cases was used to assess the presence or absence of the HPV16 E2 gene, concerning the HPV16 E6 gene. Genomic DNA of test samples (50ng) was PCR amplified for HPV16 E2 and E6 in a 25μl reaction mixture containing 10mM Tris-HCl (pH 8.4), 50mM KCl, 1.5mM MgCl_2_, 125μM of each dNTPs (dATP, dGTP, dCTP, dTTP), 5pmol of oligonucleotide primers for either full-length HPV16 E2 or HPV16 E6 and 0.5U AmpliTaqGold DNA polymerase (Applied Biosystems, USA). The amplification was performed with an initial denaturation at 95°C for 4 min, polymerization for 35 cycles of denaturation at 95°C for 30sec, annealing at 55°C for 30sec and extension at 72°C for 1min, which was extended for 5min at the final cycle (Applied Biosystems). The densitometric ratio of E2 and E6 amplicons was measured on AlphaDigiDoc using Alpha Ease FC version 4.1.0 (Alpha Innotech Corporation, USA) to determine the physical state of HPV16 for each sample. Densitometric ratios of E2: E6 amplicons of all clinical samples were normalized to E2: E6 ratio of vector-free HPV16 plasmid (a kind gift from Prof. H. zur Hausen, DKFZ, Germany) which was used as a reference for a pure episomal form of HPV16 genome and helped to normalize the variations in PCR efficiencies. DNA from SiHa cells (Procured from American Type Culture Collection, USA) was used as a control for fully integrated DNA. The E2: E6 ratio in clinical samples with reference to the plasmid control was calculated by the following formula:

#### Normalized E2: E6 ratio of clinical samples = (IDV E2: IDV E6) samples /(IDV E2: IDV E6) plasmid

where IDV indicates integrated densitometric values of DNA band of HPV16 E2 amplicon (IDV) or HPV16 E6 (IDV) amplicon of the plasmid and sample DNA. An E2: E6 ratio with a value of 0 represented completely integrated HPV16 genome and value of 1 or higher represented predominantly episomal viral genome, whereas values > 0 and < 1 indicated a mixed form of HPV16 DNA.

### pSTAT3 immunoblotting in proteins isolated from cervical tumor tissues

Isolation of total cellular proteins from fresh biopsies and immunoblot analyses were performed using pSTAT3 (Y705) and pSTAT3 antibodies (BD Biosciences, USA) as described previously [29]. Membranes were re-probed for β-actin as an internal loading control. The quantitative densitometric analysis of the bands was performed using Alpha Ease FC version 4.1.0 (Alpha Innotech). The expression level of proteins was quantitated on an arbitrary scale with respect to the β-actin expression where Strong (+++)–>50%; Medium (++)– 10–50%; Weak (+)–<10% of β-actin expression; and Nil (–)–not-detectable.

### Statistical analysis

The data analysis was performed using the statistical software SPSS version 17 and SigmaPlot v14.0 (Systat Software, Inc.). To determine the mean and median values of continuous variables and standard errors of means, descriptive statistics were used. As the distribution of viral load and E2: E6 ratio significantly departed from approximate normality, non-parametric tests were used to compare the distribution of study measurements across the study groups. Mann Whitney U test was used to compare the distribution of viral load and physical state of HPV16 along with the status of pSTAT3 expression between different disease groups. *p* values of <0.05 were considered statistically significant. Association between the level of pSTAT3 expression, viral load, and physical status of HPV16 among different categories of tissues from precancer and cancer lesions was examined by non-parametric Spearman’s rank-order correlation coefficient. Cervical disease groups were converted to categorical variables based on their increasing severity. pSTAT3 expression was evaluated on 4 point intensity scale as mentioned above, whereas, viral load and physical state (E2: E6 ratio) were evaluated as continuous variables. These variables were examined for significance and strength of the relationship between study parameters in overall study samples and between test groups. To evaluate the association between pSTAT3, viral load, and physical state of HPV16 One Way Analysis of Variance (ANOVA) on ranks were performed using Kruskal-Wallis test, and multiple pairwise comparison was performed using the Tukey Test. Chi-square test was performed between two levels of STAT3 expression with respect to the three different physical states of the HPV16 genome where applicable.

## Results

**Fig 1:**
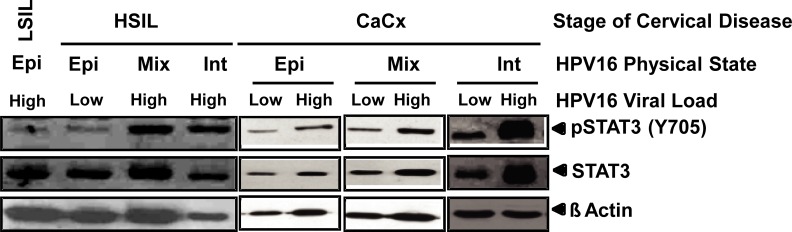
Status of pSTAT3, along with the physical state and viral load of HPV16 in different cervical pre-cancer and cancer tissues. Representative photograph showing levels of active pSTAT3(Y705) and total STAT3 in HPV16 positive cervical lesions. Cellular proteins (40μg/lane) derived from representative cervical tissues with indicated physical state of HPV16 genome (episomal- Epi; mixed–Mix; or integrated–Int) and viral load either lower (Low) or higher (High) than the median value of respective stage of disease were examined for pSTAT3 (Y705) and STAT3 expression by immunoblotting. Blots were stripped and re-probed with β-actin to equate the variations in cellular protein input.

Level of pSTAT3 (Y705) was analyzed by immunoblotting in a total of 130 HPV16 positive cases comprising 60 pre-cancer (LSIL– 30; HSIL– 30) and 70 cancer tissues and a correlation was examined with respective viral load and physical state of HPV16 genome from corresponding cervical lesions (**Fig 1**).

### Association between pSTAT3 expression and HPV16 viral load

**Fig 2:**
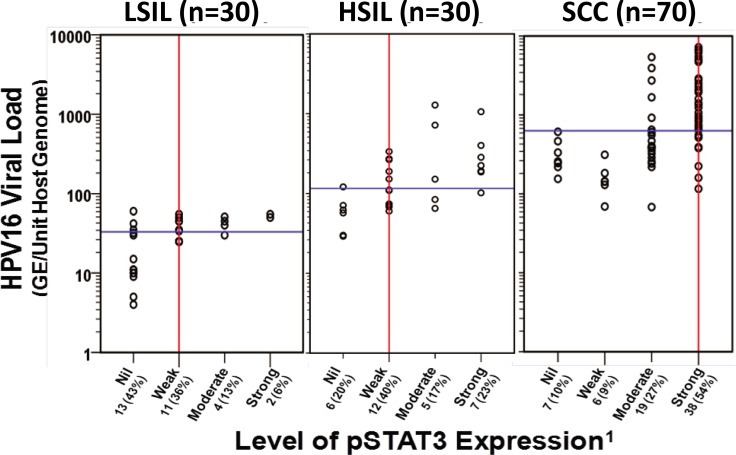
Distribution of HPV16 viral load and pSTAT3 levels in cervical pre-cancer and cancer lesions. Each circle represents individual LSIL, HSIL, or cancer tissues (SCC) with respective viral load and pSTAT3(Y705) level as determined by real-time PCR and immunoblotting. Viral load values are normalized per cell genome equivalent as described in Methods. ^1^Arbitrary level of STAT3 expression in immunoblotting: Strong = (+++), Moderate = (++), Weak = (+), Nil/undetectable = (-). The vertical red line and horizontal blue line represent median values of STAT3 expression and viral load in tissues of each disease stage, respectively. GE, genome equivalents.

Distribution of samples concerning their viral load and level of pSTAT3 expression in LSIL, HSIL, and cancer (SCC) groups is presented in **Fig 2**. The median values for HPV16 viral load increased with disease severity from LSIL (33 GE/unit host genome) to HSIL (116 GE/unit host genome) and were the highest in cancer tissues (629 GE/unit host genome). Both LSIL and HSIL tissues, in general, had a weaker pSTAT3 expression along with lower viral load compared to cancer tissues which expressed stronger pSTAT3 and had consistently higher HPV16 viral load (p<0.001). Analysis of viral load and pSTAT3 level within different stages of disease demonstrated higher viral loads in samples that expressed moderate or strong pSTAT3 expression (**Table 3**). In LSIL, the moderate or strong expression of pSTAT3 was associated with the higher median copy number of HPV16 (47.5 GE/unit host genome) in comparison to the LSIL that had a weak or undetectable pSTAT3 expression (27.5 GE/unit host genome; *p*-value < 0.05). Likewise, in HSIL, the median copy number associated with moderate or strong pSTAT3 expression (207.5 GE/unit host genome) was 3-fold higher than a median viral load of cases with nil or weak pSTAT3 expression (73.5 GE/unit host genome; *p*-value < 0.01). In cancer tissues, the strong pSTAT3 expression was associated with higher median viral copy number (845 GE/Unit host genome) than cases with weak pSTAT3 expression (219 GE/Unit host genome; *p*-value < 0.001) (**Table 3**). The results demonstrate a progressive increase in viral copies with higher STAT3 activity that increased with the increasing severity of cervical lesions.

**Table 3:** Distribution of HPV16 viral load in HPV16 positive cervical precancer and cancer lesions with respect to the level of pSTAT3 expression.

Tissue Type	Parameter	HPV16 Viral Load (GE/Unit Host Genome)		*p* value^a^
		pSTAT3 Nil / Weak	pSTAT3 Moderate / Strong	
**LSIL (n = 30)**	Median	27.5	47.5	**<0.05**
	Mean **±** SE	28.0 **±** 3.3	45.1 **±** 3.7	
	N	24	6	
**HSIL (n = 30)**	Median	73.5	207.5	**<0.05**
	Mean **±** SE	119.4 **±** 55.0	395.1 **±** 117.1	
	N	18	12	
**SCC (n = 70)**	Median	219	845.0	**<0.001**
	Mean **±** SE	251.4 **±** 41.7	1766.1 **±** 260.6	
	N	13	57	

LSIL—Tissues derived from Low grade Squamous Intraepithelial Lesions; HSIL—Tissues derived from High grade Squamous Intraepithelial Lesions; SCC—Tissues derived from Squamous Cell Carcinoma; SE–Standard Error

^a^*p*-value between pSTAT3 (Nil/Weak) vs. pSTAT3 (Moderate/High) in each disease stage as determined by Mann-Whitney U test. Bold type refers to statistically significant *p*-values

### Association between the expression of pSTAT3 and integrated HPV16 genome in pre-cancer and cancer lesions

**Fig 3:**
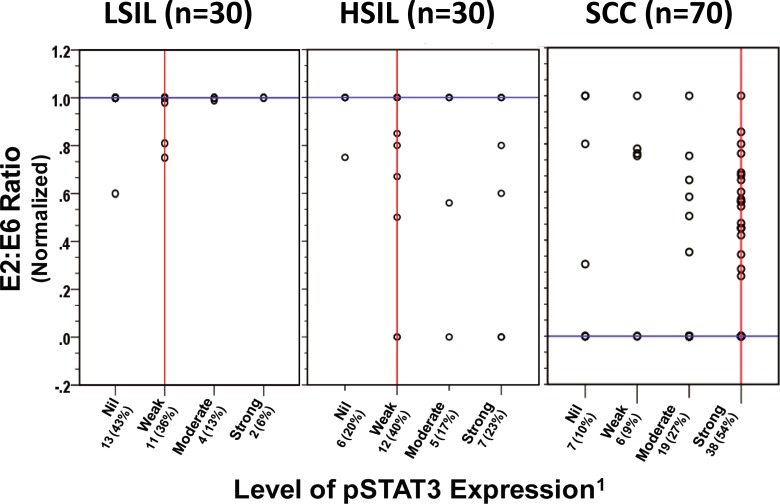
Analysis of the physical state of HPV16 genome and level of active pSTAT3(Y705) in cervical pre-cancer and cancer cases. Distribution of normalized HPV16 E2: E6 ratio and level of pSTAT3 expression in cervical pre-cancer (LSIL and HSIL) and cancer cases (SCC). Each circle indicates an individual case. Normalized E2: E6 ratios were determined by PCR and calculated as described in Methods. Normalized E2: E6 ratio = 0 represents predominantly integrated HPV16 genome, value = 1 represents predominantly episomal viral genome, and values between 0 and 1 indicate a mixed form of HPV16 genome. ^1^Arbitrary level of pSTAT3 expression in immunoblotting: Strong = (+++), Moderate = (++), Weak = (+), Nil/undetectable = (-). The vertical red line and horizontal blue line represent median values of pSTAT3 expression and E2: E6 ratio in each disease group, respectively.

Next, we examined the association of the physical state of HPV16 genome classified as predominantly episomal, integrated; or mixed form (concomitant presence of both episomal and integrated) which was identified using normalized HPV16 E2: E6 ratio, with levels of pSTAT3 in cervical pre-cancer and cancer lesions. Interestingly, pre-cancer tissues harboring episomal form demonstrated similar lower levels of pSTAT3 expression as in cancer tissues harboring episomal form, whereas the intensity of pSTAT3 was found to be higher in pre-cancer and cancer lesions harboring either mixed or integrated form of HPV16 genome (**Fig 1)**. Distribution of normalized E2: E6 ratio on the basis of pSTAT3 expression pattern, demonstrated that in most of the LSIL tissues where the HPV16 genome was present in the episomal form (median E2: E6 ratio—1.0), pSTAT3 expression was found to be either absent or weak. On the other hand, a significant number of HSIL and cancer tissues harboring either mixed or integrated form of HPV16 genome showed the elevated expression level of activated STAT3 (Median normalized E2: E6 ratio– 0; Median pSTAT3 expression–Strong) (**Fig 3**). Evaluation of the physical state of HPV16 and pSTAT3 expression in tissues from LSIL and HSIL cases, however, did not reveal any association between pSTAT3 expression with the mixed or integrated state as lesions harboring mixed or integrated HPV16 genome expressed variable amounts of pSTAT3 (**Table 4**). On the other hand, stratification of total cancer cases with respect to HPV16 physical state and level of pSTAT3 expression revealed a significant association (*p* values <0.05 and <0.001, respectively) between high pSTAT3 expression and integrated or mixed state of HPV16 genome.

**Table 4:** HPV16 physical status and level of pSTAT3 expression in cervical precancer and cancer lesions.

Tissue Type	pSTAT3 Levels	Physical State of HPV16 Genome			Total (%)	*p-*value*
		Episomal (%) (E2:E6 ≥ 1.0)	Mixed (%) (0 < E2:E6 > 1.0)	Integrated (%) (E2:E6 = 0)		
**LSIL** **(n = 30)**	Nil/Weak	21 (70)	3 (10)	-	24 (80)	0.361
	Moderate/Strong	6 (20)	-	-	6 (20)	
**HSIL**	Nil/Weak	11 (36)	5 (17)	2 (7)	18 (60)	0.603
**(n = 30)**	Moderate/Strong	6 (20)	3 (10)	3 (10)	12 (40)	
**SCC**	Nil/Weak	3 (4)	5 (7)	5 (7)	13 (18)	**<0.04**
**(n = 70)**	Moderate/Strong	2 (3)	24 (35)	31 (44)	57 (82)	
**Total** **(n = 130)**	**Nil/Weak**	**35 (27)**	**13 (10)**	**7 (5)**	**55 (42)**	**<0.001**^**a**^
	**Moderate/Strong**	**14 (11)**	**27 (21)**	**34 (26)**	**75 (58)**	

LSIL—Tissues derived from Low-grade Squamous Intraepithelial Lesions; HSIL—Tissues derived from High-grade Squamous Intraepithelial Lesions; SCC—Tissues derived from Squamous Cell Carcinoma; SE–Standard Error

^a^Pearson Chi-square p<0.001between two levels of STAT3 expression concerning the three different physical status of HPV16.

*Bold type refers to statistically significant *p* values.

### HPV16 viral load, its physical state, and level of active pSTAT3 are mutually related

Further, analysis of pSTAT3 expression with respect to the differential viral load in different physical states of the HPV16 genome revealed lower levels of pSTAT3 expression in samples with lower viral load and episomal form as compared to cervical lesions with either higher viral load and/or with integrated or mixed form of HPV16 genomes (**Fig 1**). Cumulative data presented in **Table 5**revealed that LSILs with HPV16 episomes and having moderate or strong pSTAT3 harbored higher copy numbers of HPV16 genome (Med—25 vs. 47.5 GE/unit host genome; *p*-value = 0.012) whereas HSIL with episomal HPV16 showed an increased pSTAT3 expression was associated with higher viral copies (Median—68 vs. 126.5 GE/unit host genome; *p*-value = 0.01). However, no difference was observed in HPV16 viral load with respect to pSTAT3 expression in LSIL and HSIL cases with integrated or mixed HPV16 genome. On the other hand, cancer tissues where the HPV16 genome existed as mixed or integrated physical form showed a strong association between increased pSTAT3 and elevated viral loads (*p*-value<0.01).

**Table 5:** Correlation between active pSTAT3 with physical state of HPV16 and its viral load in different disease grade of cervical cancer.

Stage of Disease	Active pSTAT3 Expression Level	HPV16 Physical State	HPV16 Viral Load (GE/Unit Host Genome)		*p*-value
			Median	Mean ± SE	
**LSIL**	Nil/Low (n = 24)	Episomal (n = 21)	25	27.3 ± 3.0	
		Mixed (n = 3)	45	34.7 ± 12.9	
		Integrated (n = 0)	-	-	
	Moderate/ Strong (n = 6)	Episomal (n = 6)	47.5	45.17 ± 3.7	**0.012**^**a**^
		Mixed (n = 0)	-	-	NA^**b**^
		Integrated (n = 0)	-	-	NA^**c**^
**HSIL**	Nil/Low (n = 18)	Episomal (n = 11)	68	64.9 ± 6.7	
		Mixed (n = 5)	152	198.0 ± 45.0	
		Integrated (n = 2)	227	227.0 ± 38.0	
	Moderate/ Strong (n = 12)	Episomal (n = 6)	126.5	136.1 ± 25.75	**0.010**^**a**^
		Mixed (n = 3)	1059	875.1 ± 302.9	0.071^**b**^
		Integrated (n = 3)	400	435.0 ± 154.8	0.800^**c**^
**SCC**	Nil/Low (n = 13)	Episomal (n = 3)	155.00	278.7 ± 168.5	
		Mixed (n = 5)	183.00	235.4 ± 61.3	
		Integrated (n = 5)	245.00	251.0 ± 34.0	
	Moderate/ Strong (n = 57)	Episomal (n = 2)	92.00	92.00 ± 24.000	0.4^**a**^
		Mixed (n = 24)	2637.00	3098.29 ± 484.322	**<0.001**^**b**^
		Integrated (n = 31)	559.00	843.90 ± 116.970	**0.001**^**c**^

^**a**^episomal vs episomal

^**b**^mixed vs mixed

^**c**^integrated vs integrated between nil/weak and moderate/strong pSTAT3 expression within each disease stage; *p*-value determined by Mann-Whitney U test, NA- not applicable due to the zero value in particular category.

Since this being a multifaceted study comparing the level of pSTAT3 with viral load and physical state of HPV16 in different grades of cervical neoplastic disease representing cancer progression, correlation of these variables was statistically examined by non-parametric test. Assessment of association between lesion grade, viral load, E2: E6 ratio and pSTAT3 was performed using Spearman’s rank correlation coefficient which revealed a positive correlation between lesion grade, viral load and pSTAT3 with highly significant *p*-values (**Table 6**). On the contrary, a strong negative correlation existed between E2: E6 ratio and lesion grade, viral load, or pSTAT3 expression. A Kruskal-Wallis H test showed that there was a statistically significant difference in the median values among the groups which is greater than it would be expected by chance (H = 425.2 with 3 degrees of freedom; P<0.001). Similarly, pairwise multiple comparisons between lesion grade, viral load, E2: E6 ratio and pSTAT3 showed highly significant *p*-values except in the case of pSTAT3 vs. lesion grade (**Table 7**).

**Table 6:** Assessment of correlation between pSTAT3 with HPV16 viral load and E2: E6 Ratio in HPV16-positive cervical tissues (n = 130).

Variables		Viral Load	E2: E6 Ratio	pSTAT3
**Lesion grade**	**ρ**	**0.810**	**-0.710**	**0.518**
	*p-****value***	*0*.*0000002*	*0*.*0000002*	*0*.*000000000215*
**Viral Load**	**ρ**		**-0.691**	**0.696**
	*p-****value***		*0*.*0000002*	*0*.*0000002*
**E2:E6Ratio**	**ρ**			**-0.474**
	*p-****value***			*0*.*0000000163*

**ρ**- Spearman’s rank-order correlation coefficient

**Table 7:** Pairwise multiple comparison by Tukey Test between different study variables.

Comparison	Difference of Ranks	q score	*p*-value
Viral Load vs. E2:E6 Ratio	49304.5	28.780	<0.001
Viral Load vs. Lesion grade	28076.5	16.389	<0.001
Viral Load vs. pSTAT3	23925.0	13.965	<0.001
pSTAT3 vs. E2:E6 Ratio	25379.5	14.814	<0.001
pSTAT3 vs. Lesion grade	4151.5	2.423	0.317
Lesion grade vs. E2:E6 Ratio	21228.0	12.391	<0.001

## Discussion

HPV viral load and physical state of the viral genome are important determinants of HPV infection, which influence the tumorigenic transformation of normal cervical epithelium and progression of the disease [2,4,41,42]. Our earlier observations showed elevated STAT3 signaling in HPV-infected precancer and cancer lesions [43] and its functional contribution in cervical carcinogenesis [43]. In the present study, viral load and physical state of infecting virus were collectively examined for any correlation with expression of pSTAT3 to assess whether these are related events during the progression of cervical carcinoma.

Even though E2: E6 ratio is a gross test to evaluate integration and there are more sophisticated assays like real-time quantitative PCR, APOT or NGS, it is sufficiently indicative to infer a potential loss of E2 expression in the sample. Further, the E2 PCR characteristically covers full length HPV16 E2 region (amplicon size– 1139bp) which eliminates the chances of falsely interpreting integrated HPV as episomal. The full length E2 primers used and the PCR based assay to assess physical state was developed and validated by our laboratory previously [37][2]. The E2 primers produced a considerably long PCR product (1139bp) that was not amenable to real-time quantitation. This restricted us to stay with the conventional end-point PCR technique. The gross E2: E6 ratios derived from densitometric values of each samples post PCR were normalized to E2: E6 ratio obtained from densitometric values of concurrently amplified HPV16 plasmid which was taken as control and represented the episomal state of the HPV genome.

The present study specifically focused on HPV16 genotype. Viral load of HPV16 but not the HPV type 18 was demonstrated to express the highest predicted value (96% specificity and 88% sensitivity) in an earlier study [44]. In a similar study on HPV16, HPV18, HPV31, and HPV45, a clear dose-response pattern relationship between HPV16 DNA loads and infection clearance was observed, whereas, other types failed to show such relationship [45]. Therefore, in the present study, tissues positive for other types of HPV infections, with or without HPV16 infection, that could potentially introduce confounding variables were excluded from the analysis to maintain the homogeneity of the study.

The present investigation revealed that expression of pSTAT3(Y705) within different stages of the cervical carcinogenesis (LSIL, HSIL or SCC) individually as well as collectively, was associated with the degree of HPV16 viral load. A recent study has experimentally shown that loss of STAT3 severely impacts episomal maintenance of HPV18 in undifferentiated keratinocytes [36]. The study highlighted the role of viral oncoprotein E6 in HPV18 viral genome amplification, whereas, a high level of E6 was reported in invasive cervical cancers that had episomal HPV16 [46]. In an analogous system of virally-induced hepatocellular carcinoma, the role of active STAT3 has been suggested in HCV replication [47]. STAT3-mediated molecular mechanisms that operate and control HPV16 viral load in infected lesions are, however, unknown. Earlier studies from our laboratory showed expression of HPV16 E6 forms a positive regulatory loop with STAT3 [43,48] and other stemness-associated markers, GLI and Hes1, to promote oncogenic activity [34,49]. This could be likely via selective overexpression of E6 in some of the cells positive for HPV16, if not all cells of the lesions, which could lead to increase pSTAT3 level in patients with severe disease (and integrated genomes). HPV E6 has been implicated in activation of EGFR and expression of IL-6 and Oncostatin M [36,50–52], which collectively work as STAT3 upstream activators. However, any direct action of increased pSTAT3 on the integration of HPV16 in the host genome in either cancer or precancer tissues, is not known as yet. The present investigation only provides evidence of a positive correlation between these two events, and is suggestive of a potential interlink. Due to the lack of an appropriate experimental model for HPV16 integration, the possibility of evaluating the direct influence of active pSTAT3 on HPV16 integration events or vice versa remains unexplored. Given these observations, our data provide first clinical evidence that this phenomenon could also operate in an *in vivo* situation in HPV16-infected cervical precancer and cancer lesions.

A comparative analysis of pSTAT3 expression with HPV16 physical state further demonstrated that in most of the LSIL cases where the HPV16 genome was present in predominantly episomal form, STAT3 activation was weak. On the other hand, tissues of HSIL and cancer lesion harboring either mixed or predominantly integrated form of the HPV16 genome showed an elevated level of active pSTAT3. This correlation sustained in cancer tissues harboring episomal form demonstrated a low level of active STAT3. Our data, therefore, indicated a possible association between STAT3 activation and integration of the viral genome in HPV16 infections. The mechanism by which STAT3 mediates its effect on the process of integration, which is considered as an opportunistic and random event during carcinogenesis, is not known. In such a scenario, an indirect mechanism, such as the induction of genomic instability [26] by which STAT3 could influence integration events, cannot be ruled out.

Taken together, our study shows for the first time a clinical correlation between pSTAT3, viral copy number, and physical state of the HPV16 genome during cervical disease progression. Independent of its direct role in epithelial carcinogenesis [31], STAT3 may be involved in existence as well as pathological manifestation of HPV16 genome and could be utilized as possible therapeutic, diagnostic and prognostic target for potentially-progressive cervical cancer and pre-cancer lesions.

## Abbreviations

HPV: human papillomavirus
HSIL: high grade squamous intraepithelial lesions
LSIL: low grade squamous intraepithelial lesions
SCC: squamous cell carcinoma
pSTAT3: phospho-tyrosine705-signal transducer and activator of transcription
URR: upstream regulatory region
